# Improving communication during damage control surgery: a survey of adult major trauma centres in England

**DOI:** 10.1308/rcsann.2024.0087

**Published:** 2024-10-22

**Authors:** EN Patton, I Lisagors, I Tyrrell-Marsh, S Agarwal, LV Wee, A Darwish, SR Smith

**Affiliations:** Manchester University NHS Foundation Trust, UK

**Keywords:** adult, checklist, trauma centres, surveys and questionnaires

## Abstract

**Introduction:**

Deficiencies in non-technical skills can severely impede the functioning of teams in high-intensity scenarios, such as in damage control surgery for the critically injured trauma patient. Truncated preoperative checklists, modified from the standard World Health Organization preoperative checklist, and situational reporting at intervals during surgery are long-established practices in the military, and are recommended in the National Health Service guidelines on major incidents. These tools allow the multiprofessional team to create a shared mental model of the anaesthetic and operative plan, thereby improving team efficiency. Our aim was to establish whether adult major trauma centres in England are using truncated preoperative checklists and situational reporting for damage control surgery.

**Methods:**

An online survey was devised and distributed via the national programme of care for trauma in November 2020.

**Results:**

Responses were received from all 23 adult major trauma centres in England. Nine centres (39.1%) reported using a truncated preoperative checklist for damage control surgery albeit in a variety of formats. Common components were blood products received and/or available, presence of allergies, tranexamic acid and antibiotic administration, availability of viscoelastic tests, equipment required, availability of cell saver, role allocation and reference to other personnel needed, and discussion of the plan. Twelve centres (52.2%) have formal policies in place for situational reporting. Again, these were in multiple formats but all focused on patient physiology to direct surgical planning.

**Conclusions:**

We have identified key components to advanced communication aids for damage control surgery, providing a foundation on which other major trauma centres can build their own versions of these potentially lifesaving tools.

## Introduction

Damage control describes a systematic approach to the management of exsanguinating trauma patients. Damage control resuscitation involves haemostatic resuscitation with blood products to restore circulating volume, optimise oxygen delivery, correct coagulopathy and warm the patient. It occurs concurrently with damage control surgery, which aims to gain control of bleeding and contamination, and minimise further ischaemia.^1,2^ Damage control prioritises correcting physiology over restoration of anatomy to prevent or reverse coagulopathy, hypotension, hypothermia and acidosis.^1,3^ Temporising surgical manoeuvres are utilised, such as shunting large blood vessels or stapling off open bowel, to permit the patient to repay their metabolic debt in an intensive care environment before returning to theatre for definitive surgery.^2,3^ The principles of damage control are distinct from those employed to manage the elective surgical patient and the working environment is markedly more pressured.

The role of human factors in the functioning of teams in demanding circumstances is well documented.^4–7^ Individuals in these situations can become task-focused and unable to maintain situational awareness.^8,9^ They may well be working outside their comfort zones.^10^ As a result, significant barriers to effective teamwork and communication can emerge.^11^ Communication aids ensure delivery of key facts in a set format without extraneous information and improve team functioning.^7^ In damage control, communication aids consist of:

• Truncated preoperative checklists for damage control scenarios to remove immaterial information from elective World Health Organization (WHO) checklists, focusing instead on resuscitation and surgical planning based on physiological parameters^12–14^
• Situational reporting involving regular intraoperative updates of patient physiology, surgical progress and the ongoing plan – This is designed to create a shared mental model of the operative and resuscitative goals throughout the course of the procedure.^12,13,15,16^
• Modified sign-out checklists highlighting other injuries requiring attention and ongoing resuscitation needs, forming a crucial part of the handover to intensive care colleagues, ensuring seamless care^12^

The high concentration of major trauma dealt with by the UK’s Defence Medical Services in Camp Bastion, Afghanistan, led to the development of tools to improve communication during damage control.^12,13^ These consisted of a truncated WHO preoperative checklist on arrival to theatre (‘snap brief’), situational reporting (‘sit reps’) and a modified sign-out at the end of surgery.^12,13^ The ‘snap brief’ consisted of:^17^

• Starting the clock – This is considered ‘time zero’.
• The surgeon should confirm the correct patient as well as the main injuries found on clinical and imaging findings, and state the surgical plan including timescale.
• The anaesthetist should state:
  - o temperature;
  - o blood pressure, blood gas, blood products transfused so far and current rate of infusion;
  - o coagulopathy (thromboelastography/thromboelastometry results if available);
  - o any other clinical problems that have been recognised or are evolving.

The format of situational reporting utilised in Camp Bastion followed the mnemonic TBCS and an alternative format utilised by the military is TSTACKP.^13,18^ These are outlined in Table 1. The sign-out utilised in Camp Bastion was performed in the presence of the accepting team (usually critical care) and consisted of:^13^

• The surgeon describing the injuries found on examination, on radiology and at operation, plus a description of the surgery performed, any injuries not treated and the plan for further surgery
• The anaesthetist describing the patient’s physiology at the start and end of the procedure with specific reference to any critical events, plus the volume of blood given as well as the most recent thromboelastometry/thromboelastography and blood gas results

**Table 1:** Examples of formats of situational reporting

TBCS^13^	TSTACKP^18^
• Every 10–30 minutes during surgery, especially if change in physiology or surgical plan • The anaesthetist should state: o **T**ime since start of procedure and **T**emperature o **B**lood pressure, **B**lood products given and rate of infusion, and **B**lood gas o **C**lotting results and temperature (**C**old) o Any developing problems • The surgeon should state: o **S**urgical progress (phase, e.g. vascular control, resuscitative or therapeutic packing etc) o New developing problems/findings o **S**urgical plan	• **T**ime from knife to skin (KTS) • **S**ystolic blood pressure • **T**emperature • **A**cidosis (i.e. blood gas results) • **C**lotting and blood products transfused • **K**it available/unavailable (plus limits of surgery/consideration of futility) • **P**lan and **P**rogress

NHS England guidance on the management of major incidents and mass casualty events recommends the use of truncated preoperative checklists and situational reporting intraoperatively.^19^ However, it does not specify what format these should take.

In 2012, 26 major trauma networks were established across the UK to deliver specialist trauma care and rehabilitation.^20^ Major trauma centres tend to be larger, inner-city hospitals with the infrastructure and surgical capability to provide lifesaving surgery across multiple specialties. The extent to which the communication aids utilised in military practice have been adopted into the civilian healthcare environment is unclear. We aimed to examine practice across England, through a national survey of adult major trauma centres, to establish whether formal systems are used to aid communication during damage control surgery.

## Methods

An online survey (www.smartsurvey.co.uk) was devised and distributed by email, via the national programme of care for trauma, to each of the clinical and operational leads in the 23 adult major trauma centres in England. The national programme of care provides leadership and oversight of the development and delivery of a comprehensive work programme for trauma, and as such, was able to contact the leadership teams easily. The survey was distributed in November 2020.

Where no response was received within 30 days, individual emails were sent to major trauma clinical leads or directors in order to prompt a response. Where appropriate, clinical leads delegated completion of the questionnaire to a surgeon or anaesthetist working in the major trauma service, for instance if the clinical lead did not work in theatres. The management of paediatric trauma, especially in those aged under 12 years, differs to that of adults so the focus of our survey was on adult care.

**Figure 1:**
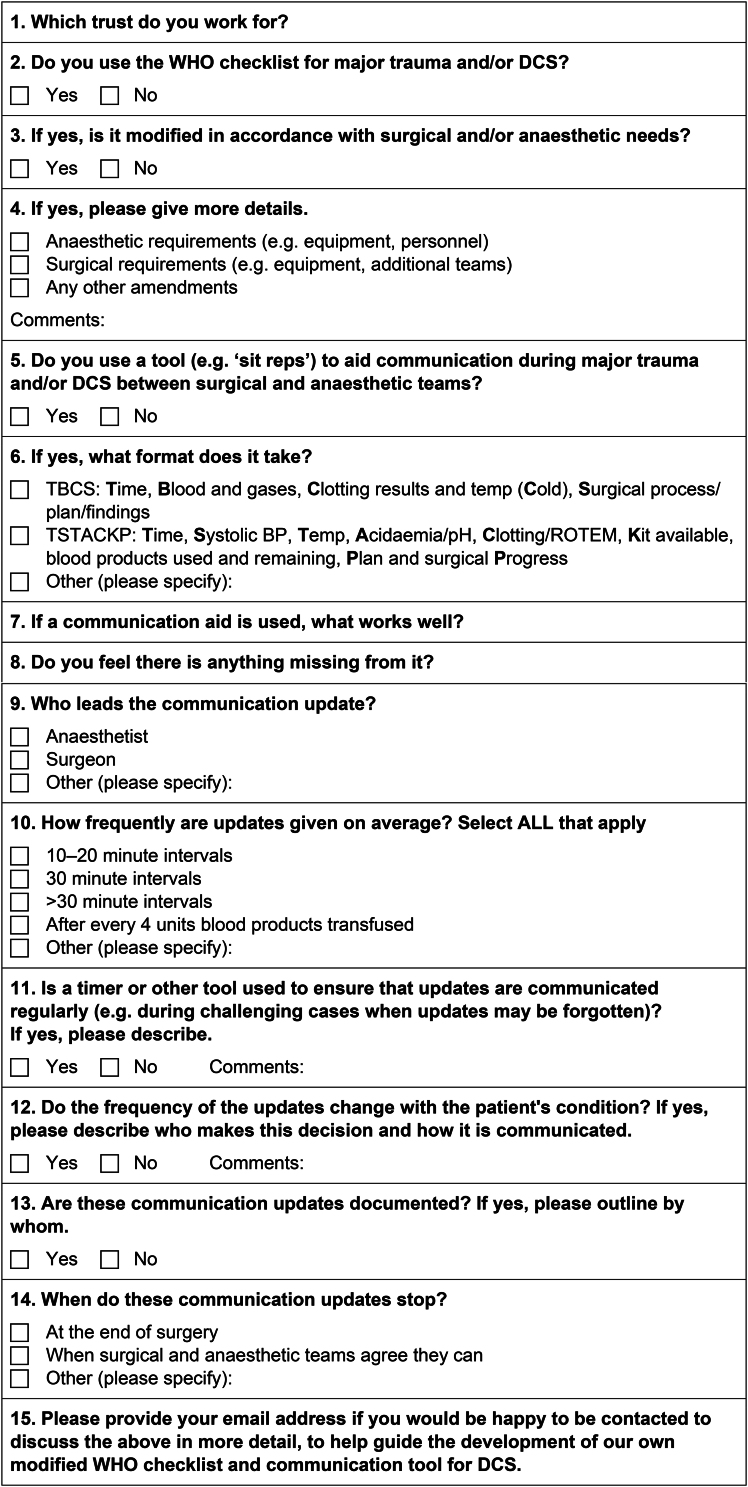
Online survey on use of truncated preoperative checklists and situational reporting during damage control surgery (DCS) distributed to adult major trauma centres in England

The questionnaire was designed by three of the authors: a consultant surgeon (SRS), a consultant anaesthetist (IL) and an anaesthetist in training (ENP). The questions were chosen based on understanding of the available literature, a desire to explore current practice and potential limitations of communication aids being used. The questionnaire consisted of 14 questions relating to the use of truncated WHO preoperative checklists and situational reporting for damage control surgery, as outlined in Figure 1. The survey was designed to be narrative with open questions to permit respondents to describe the practice in their institution. Questions on the use of a modified sign-out were not included, which was an inadvertent omission. There was no pilot phase to the project. There was the option for centres to email us their policy (where it existed) and we encouraged centres to do this if we contacted them directly.

Ethical approval was not required for this project as it was a service evaluation with no patient data included.

## Results

Responses were obtained from all 23 adult major trauma centres in England between December 2020 and November 2021. Multiple attempts at contact were needed for some institutions.

### Do you use the WHO checklist for damage control surgery? Is it modified?

Respondents indicated that 22 of the 23 adult major trauma centres use a preoperative checklist in damage control surgery. Thirteen (56.5%) are using the standard WHO checklist prior to damage control surgery while nine (39.1%) have a truncated checklist. Every checklist was different (Table 2).

**Table 2:** Components of preoperative checklists and number of centres using them

		Number of centres (*n*=9)
Patient-related elements	Blood products given and/or available	8
	Allergies	6
	Tranexamic acid administration	5
	Thromboelastography/thromboelastometry results	3
	Antibiotics given	3
	Consent form completed	3
	Venous/arterial blood gas results	2
	Temperature	2
	Systolic blood pressure	2
Personnel-related elements	Surgical plan	7
	Teams present and/or additional personnel required	4
	Role allocations (e.g. team leader, blood runner, scribe)	3
Equipment-related elements	Equipment needed	7
	Cell saver requested	4
	Equipment and swabs already in the patient (e.g. from resuscitativeendovascular balloon occlusion of the aorta or resuscitative thoracotomy)	3
	Fluid warmer set up/patient warming considered	2
	Intensive care bed requested	1
	Diathermy set up	1
	Need for spinal precautions	1

Although we did not ask specifically, five centres volunteered that they have a team brief, consisting of a handover from the emergency department trauma team leader. All these centres used the ATMIST abbreviation (Age, Time of injury, Mechanisms of injury, Injuries suspected, Signs, Treatments given). Four of these centres had also developed a modified preoperative checklist. One centre had embedded an initial situational report, including patient physiological status, into their preoperative checklist.

We were able to obtain the modified preoperative checklist for four of the nine centres using them. One centre with a modified preoperative checklist also used action cards as an aide-mémoire for each person as to their role. Interestingly, one centre that did not use a modified checklist did have action cards for every team member in theatre, which they shared with us. Each team member states certain parameters at the team brief. The brief included a handover from the emergency department team. These parameters are the same as those included in the modified checklists in other centres.

### Do you use situational reporting during damage control surgery to aid communication between surgical and anaesthetic teams? If yes, what format does it take?

Twelve of the adult major trauma centres (52.2%) have a formal policy for situational reporting during damage control surgery (Table 3). Seven centres (30.4%) indicated that situational reporting is used on an ad hoc basis depending on the clinicians involved and that it consequently has varying formats. Four centres (17.4%) do not use situational reporting. All major trauma centres using situational reporting reference the time since the start of the procedure, the patient’s current physiology, the surgical progress and discussion of the plan going forwards.

**Table 3:** Format of situational reporting in adult major trauma centres in England with a formal policy in place

Situational reporting format	Number of major trauma centres using situational reporting (*n*=12)
TBCS	3
TSTACKP	5
Locally-designed acronym	4

### Who leads situational reporting?

Of the 12 centres with a formal policy for situational reporting, 8 centres (66.7%) indicated that these are led by the anaesthetist. One centre (8.3%) stated that they are led by the surgeon. Three of the twelve centres (25.0%) responded that the individual leading situational reporting depends on the skill mix available, with operating department practitioners, theatre runners and major trauma consultants in a coordinating, non-clinical role all being utilised.

### How frequently is situational reporting undertaken? Does the frequency of the updates change with the patient's condition?

Eleven of the twelve centres with a formal policy (91.7%) aim to have a situational report every 10–20 minutes, with one (8.3%) stating that they aim for every 20 minutes (Table 4). Two centres (16.7%) also have a situational report after every four units of blood products transfused. Additionally, respondents indicated that situational reports should be flexible and could be triggered by the surgeon or anaesthetist following changes in the clinical condition or surgical findings. Most centres with a formal policy for situational reporting take this approach, with ten of the twelve centres (83.3%) changing the frequency of situational reports throughout the course of the operation.

**Table 4:** Timing of situational reporting in adult major trauma centres in England with a formal policy in place

Triggers for situational reports	Number of major traumacentres (*n*=12)
10–20 minute intervals	11 (91.6%)
20 minute intervals	1 (8.3%)
≥30 minute intervals	0 (0%)
After every 4 units blood products transfused	2 (16.6%)
Frequency changes with patient's condition	10 (83.3%)

### Is a timer or other tool used to ensure that situational reporting is undertaken regularly?

Two of the twelve centres with a formal policy (16.7%) indicated that they use a timer and both undertake situational reports every 10–20 minutes. Of the ten centres that do not formally use timers, two (20.0%) stated that their use is encouraged and a further two (20.0%) highlighted a timer as a missing key element given that situational reports are sometimes forgotten.

### Are situational reporting updates documented?

Two of the twelve centres with a formal policy (16.7%) responded that situational reports are documented. Both centres are recording this in the anaesthetic chart.

### When is situational reporting stopped?

When asked about when situational reports stop, seven of the twelve centres with a formal policy (58.3%) continue until anaesthetic and surgical teams agree they can stop, with the other five centres (41.7%) continuing until the end of the operation.

## Discussion

This study has shown that a third of major trauma centres in England are using a modified preoperative checklist for damage control surgery and half are using situational reporting with a formal policy. Those without a formal policy indicated that situational reporting may occur but this is personnel dependent. There is clearly wide variation in practice, the reasons for which will be multifactorial. As some centres noted in their responses, they see little exsanguinating haemorrhage so implementing a change of practice around damage control is not a high priority. Conversely, other centres have the benefit of personnel from a military background who are familiar with communication aids and are keen to adopt them into civilian practice.

A lack of formal policy and reliance on individuals to recall and implement specific ways of communicating under stress is risky. Recall has been shown to be impaired in stressful situations.^21^ Individuals are likely to forget important elements of an abbreviated preoperative checklist (or indeed of situational reporting) during damage control. Damage control surgery is a complex, highly challenging situation involving a large multiprofessional team who may lack familiarity with these scenarios.^7,10^ Failure of non-technical skills (particularly communication and teamwork) has been linked to adverse events, including death.^22–25^ Embedding formal protocols and systems into the theatre environment when it is at its most pressured is judicious. This applies not only to major trauma but also to any scenario resulting in major haemorrhage (for instance, gastrointestinal, vascular or peripartum haemorrhage) and may be applicable in complex elective surgery with potential for significant blood loss.

The WHO checklist was designed in 2009 to mitigate human error and a pilot study resulted in the in-hospital mortality rate falling from 1.5% to 0.8%.^22,26^ However, the checklist must be used properly to obtain good results.^22,27^ In damage control, there needs to be a shared mental model of the goals of resuscitation and surgery with an associated timeframe. The WHO checklist does not include patient physiology, role allocation, personnel needs or specific equipment-related points. Consequently, a different preoperative checklist is needed for damage control scenarios so that a team-wide plan based on perceived injuries and patient physiology can be created.

Our nationwide survey revealed that centres utilising a truncated preoperative checklist for damage control surgery include many of the points covered in the ‘snap brief’ of the Defence Medical Services. Nevertheless, all centres have included additional components, many of which are taken from the WHO checklist (e.g. antibiotic administration and presence of allergies).

Situational reporting has been adopted more widely than the use of a modified preoperative checklist, with 19 of 23 centres undertaking situational reporting in some format. It is not clear why this is the case. In contrast to the use of a preoperative checklist, situational reporting is not commonly performed in the elective setting. The need for situational reporting in exsanguinating haemorrhage may be born from the lack of an equivalent aide-mémoire to transfer from the elective setting. Several acronyms are used for situational reporting, with four centres using their own. However, unlike the modified preoperative checklists, there is little variation in the contents, with all centres creating a surgical plan based on patient physiology.

In the Defence Medical Services, an anaesthetist in the role of team leader (not actively resuscitating) took charge of coordinating situational reporting.^12,13^ Task-focused individuals are not best placed to regularly prompt or undertake situational reporting. As one centre highlighted in its response, like for the WHO checklist for elective operating, situational reports can be led by any appropriately trained member of staff present, including operating department practitioners, scrub staff and recovery nursing staff.

The available literature suggests that situational reports should happen within the range of every 10–30 minutes.^12,13,15,16^ NHS England’s guidance recommends every 15 minutes and suggests setting an alarm to ensure they are not missed.^19^ Interestingly, only two of the centres that are undertaking regular situational reports have a timer. Presumably in the other centres, the use of a timer on a mobile phone or clock watching is being undertaken by the situational report leader.

Triggering a situational report when new information is available, or when a set threshold of blood products has been transfused, has been recommended.^14,18^ Our results suggest that major trauma centres consider that situational reporting should be flexible, depending on patient stability, surgical progress and the need to avoid interrupting critical tasks. Respondents also indicated that decreasing the frequency of regular situational reporting can occur as the patient becomes more stable but this should be a team decision.

Only two of the twenty-three centres are documenting the contents of the situational reports. Many patients who undergo damage control surgery will have their records scrutinised through a legal process, highlighting the imperative to document the timeline contemporaneously and carefully. The recall of the attending clinicians following damage control is likely to be impaired.^21^ The consensus from the authors is that anyone who is appropriately trained as a scribe can document these scenarios. Crucially, documentation should focus on the outcome of the discussion around the plan going forwards, rather than the contents of each individual situational report. Centres must consider where best to document this. With the move towards electronic patient records, this may be on a bespoke electronic form.

On reviewing the five damage control policies that were obtained, it became clear that institutions manage damage control in different ways depending on their local expertise and resources. Some centres utilise their daily on-call staff and treat damage control similarly to a major incident scenario with action cards. Others have a designated major trauma team who are deployed to assessment and leadership roles in damage control scenarios. This demonstrates how variously institutions have adapted their systems and processes to solve the need for a different way of working during damage control. Consequently, some of the questions we asked were not entirely suitable for capturing the systems used in some institutions, especially where handover and action cards became the preoperative checklist. A pilot study may have gleaned this variation in practice and moulded the survey accordingly. Although we did request the policies from institutions, we did not follow this up if they were not supplied and it would have been useful to have obtained all the policies available.

The omission of questions relating to use of a modified sign-out is a limitation of this work. Polytrauma patients will have multiple potentially complex injuries, which may or may not have been identified in the primary survey.^28^ As many of these patients will have gone straight to theatre without computed tomography scanning,^19,29^ their plan for ongoing care will differ significantly from most postoperative patients. Apart from ongoing resuscitation and correction of physiology, they may require imaging, a secondary survey and/or urgent input from other specialties. For this reason, in addition to the usual points included in the WHO sign-out, incorporating points such as ‘known injuries left untreated’ acts as a helpful aide-mémoire for staff handing over. A modified sign-out can facilitate thorough handover and the receiving intensive care clinician should be present in theatre for it.^12,17^

**Figure 2:**
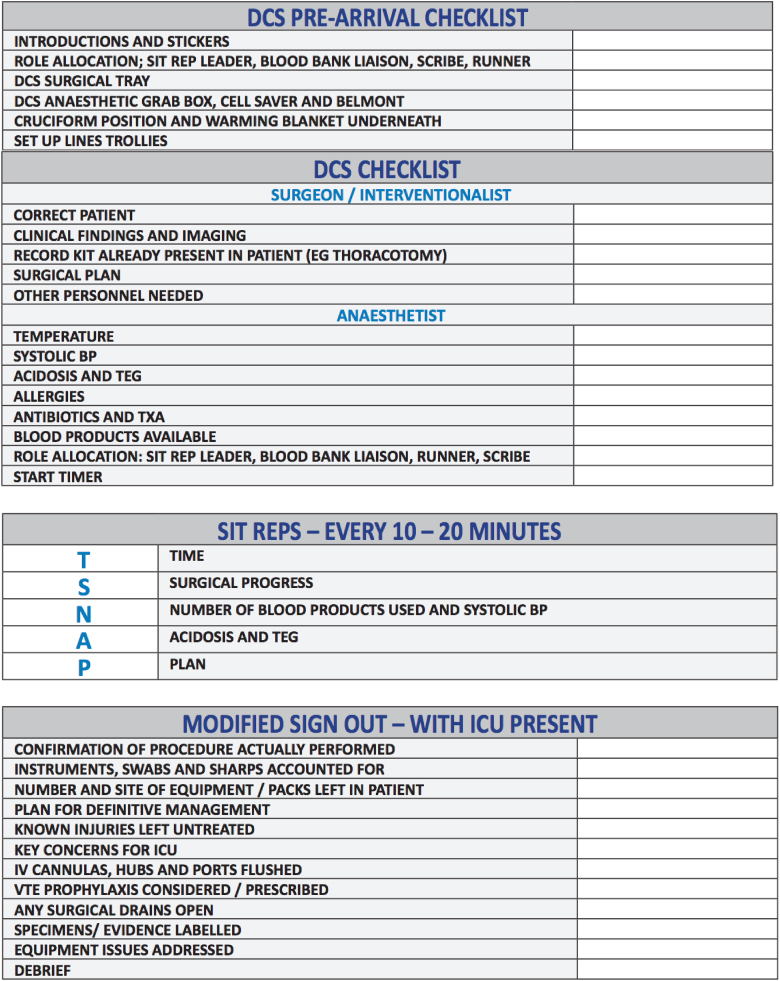
The contents of the modified preoperative checklist, situational reports and modified sign-out as implemented at Manchester Royal Infirmary major trauma centre. The preoperative checklist contains a pre-arrival section, which facilitates the theatre team’s preparations for the imminent arrival of a patient requiring damage control surgery. The situational reports take the format TSNAP.^30^ The checklists have been printed on large portable whiteboards and are located in the emergency theatres and hybrid (interventional radiology) theatres at our major trauma centre.

Following on from analysis of the nationwide survey of major trauma centres, we devised and distributed a survey to surgical, anaesthetic and theatre staff at our own major trauma centre to gather opinion on what should be included in our version of a modified preoperative checklist, situational reports and modified sign-out for damage control surgery. Expert consensus was obtained through consultation with each of the departments that could potentially be involved in damage control. The resulting proposed checklists and situational reports were systems tested through multiple rounds of in situ multidisciplinary simulation, and modifications were made based on feedback obtained and problems encountered. The result was the development of robust communication tools with stakeholder engagement from inception to implementation. Figure 2 shows the contents of our major trauma centre’s modified preoperative checklist, situational reports and modified sign-out.

This work has identified multiple areas for further research and development. We propose standardising a modified preoperative checklist, situational reports and modified sign-out across all adult major trauma centres for use in damage control scenarios. This would require ascertaining common standards and achieving expert consensus through the Delphi method^31^ while ensuring that any developed communication tools would work in the differing systems of the major trauma centres for dealing with their most critically injured patients. Furthermore, there is room for expanding the utilisation of these valuable communication tools to other high-intensity scenarios in the operating theatre, in both an elective and emergency setting.

## Conclusions

Through our nationwide survey, we have identified significant variation in practice. Most adult major trauma centres in England are not using modified preoperative checklists prior to the start of damage control surgery and only half have a formal policy for intraoperative situational reporting. By sharing good practice from across the country and reviewing the available literature, we have identified key components of modified preoperative checklists and situational reports. We hope that this will assist other major trauma centres in developing their own advanced communication aids to promote expeditious, effective decision making and teamworking during damage control surgery.
